# Safeguarding maternal health against extreme heat in India: A policy analysis of state‐level heat action plans

**DOI:** 10.1002/ijgo.70747

**Published:** 2025-12-17

**Authors:** Gabriela De Jesus Cipriano Flores, Surya Surendran, Karthika Kumar, Devaki Nambiar, Jane E. Hirst

**Affiliations:** ^1^ The George Institute for Global Health School of Public Health, Imperial College London London UK; ^2^ The George Institute for Global Health Delhi India

**Keywords:** climate change, heat, India, lactation, mothers, policy, pregnancy, temperature

## Abstract

**Objective:**

This study identifies and characterizes policy measures across Indian states to protect pregnant and lactating women from extreme heat exposure through a thematic analysis of state‐level heat and climate adaptation plans.

**Methods:**

Using content analysis, we systematically reviewed publicly available Heat Action Plans (HAPs) (*N* = 19) and, when unavailable, the State Action Plan on Climate Change (*N* = 16) from all Indian states and union territories. Strategies referencing pregnant and lactating women, either directly or indirectly (i.e., as part of a group identified as vulnerable), were extracted, categorized and coded.

**Results:**

Twelve of 35 state plans (33%) contained no recommendations for pregnant or lactating women. The remaining 23 plans listed 214 strategies, comprising 102 unique strategies (no repetitions). However, only 40 directly addressed this group. Karnataka (16), Telangana (11), and Delhi (10) had the highest number of unique strategies. Strategies were categorized using the four‐category climate adaptation framework of Chersich et al. and fell into behavior change actions (*n* = 100), structural/policy, and climate‐financing support measures (*n =* 51), health system interventions (*n =* 38), and built environment/cooling solutions (*n =* 25).

**Conclusion:**

Strategies specifically addressing pregnant and lactating women during extreme heat remain limited across Indian states, with most focusing on behavioral interventions rather than structural solutions. Critical gaps exist in 12 states with no targeted measures. Very few strategies focus on pregnant and lactating women in the HAPs. Strengthening evidence‐based, targeted adaptation policies and implementing comprehensive structural interventions are critical to protecting them from the increasing heat exposure risks.

## 1 INTRODUCTION

Extreme heat is a public health concern that is driving increasing levels of mortality and morbidity.^1^ In India, the number of heatwave days has risen significantly: the proportion of vulnerable populations (infants and adults over 65) exposed to heatwave days increased from 47% in the 1990s to 58% in the period from 2014 to 2023. This increase makes India one of the most climate‐affected countries in the world.^2^

The effects of extreme heat on health are not gender neutral,^3^, ^4^ population subgroups such as pregnant and lactating women face direct effects of extreme heat, including increased risk of miscarriage, preterm birth, low birth weight, stillbirth, intrauterine growth restriction, and birth defects.^5^

India has witnessed significant improvements in maternal and perinatal health outcomes over the past two decades, reflected in declining maternal and neonatal mortality rates.^6^ However, these gains are increasingly threatened by the rising frequency and intensity of extreme heat events. Despite advancements in maternal healthcare, India continues to report persistently high rates of preterm birth and low birth weight, both of which have been linked to heat exposure.^7^

For over two decades, the United Nations has encouraged formalized efforts to protect populations from climate change, which has fostered climate action across countries, including those facing resource constraints.^8^ This has led to the development and implementation of national adaptation policies and plans, with most countries having Nationally Determined Contributions and National Adaptation Plans.^8^ In countries with federal and decentralized systems of governance, such as India, these efforts have been extended to state or city‐based strategies known as State Action Plans for Climate Change (SAPCCs). Due to the severe impacts of extreme heat, several states and union territories (UTs) have implemented Heat Action Plans (HAPs) outlining strategies to adapt to extreme heat. These plans detail measures, technologies, and interventions adopted by communities, governments, and other stakeholders to address heat‐related risks to health and ecosystems.^9^

As countries marshal and update their climate change response plans, there is a need to craft adaptive measures and interventions specifically tailored to mitigate the impact on vulnerable subpopulations within society. Whether there are specific recommendations for pregnant and lactating women within the HAPs and SAPCCs, and what these actions are, is not clear.

Previous analyses of India's HAPs and SAPCCs have not addressed specific gaps relating to gender.^10^, ^11^, ^12^ A 2023 report on HAPs in India found them to be inadequately designed, with significant gaps in planning, financing, capacity‐building, and accountability. This report also highlighted that vulnerable groups in the document were loosely defined and not contextualized across states, which have varied.^13^ Thus, it remained unclear whether HAPs explicitly addressed the needs of pregnant and lactating women, requiring closer examination of these frameworks to identify gaps and opportunities for improvement. A global scoping review published in 2025 examined how maternal, newborn, and child health are addressed in HAPs, highlighting significant gaps in the inclusion of these vulnerable groups, including in the Indian context.^14^

Therefore, we conducted this analysis of HAPs across India to assess whether and how pregnant, lactating women, and women with young children were being considered and highlight opportunities to identify best practices and areas for improvement.

## 2 METHODS

### 2.1 Selection of resources

We conducted a desk‐based review of HAPs published by Indian states and UTs. Where HAPs were unavailable, we used SAPCCs. Documents were identified between June and July 2024 through publicly accessible websites, including the Indian Disaster Management Authority, National Centre for Disease Control portals for each state/UT, and the Global Heat Health Information Network (heathealth.info). Four additional HAPs not publicly available were obtained through professional networks. All documents analyzed are official government documents intended for public dissemination and use. Plans obtained through professional contacts with state government officials were confirmed to be public documents available for research and policy analysis purposes. No personal data or confidential information was used in this analysis. When multiple versions existed, the most recent was analyzed. Five HAPs (Bihar, Chhattisgarh, Jharkhand, Kerala, and Maharashtra) were in regional languages and translated into English using Microsoft Copilot, with translations cross‐verified for accuracy by native speakers. Complete bibliographic details and access information are provided in the reference list. Copies of documents not freely available online can be provided upon reasonable request to the corresponding author.

### 2.2 Definition of terms

We defined our population of interest as women who were either pregnant, lactating, or with young children. We use the term “lactating women” to denote the specific population vulnerable to heat effects on lactation and breast milk production, acknowledging that not all postpartum women lactate. We categorized each strategy as directly or indirectly targeted. Direct strategies explicitly targeted actions or recommendations to our study population. Indirect strategies would address them as part of a broader “vulnerable” group.

### 2.3 Data extraction

We identified and extracted all adaptive actions (strategies) that addressed our population. If a single sentence contained multiple strategies, each was extracted separately. If the same strategy was repeated in the document, we extracted each instance and added it to our database.

The strategies were extracted to Microsoft Excel. In the event of conflicts, a third reviewer read the source document, and a consensus was reached between the authors.

### 2.4 Synthesis of extracted data

**FIGURE 1:**
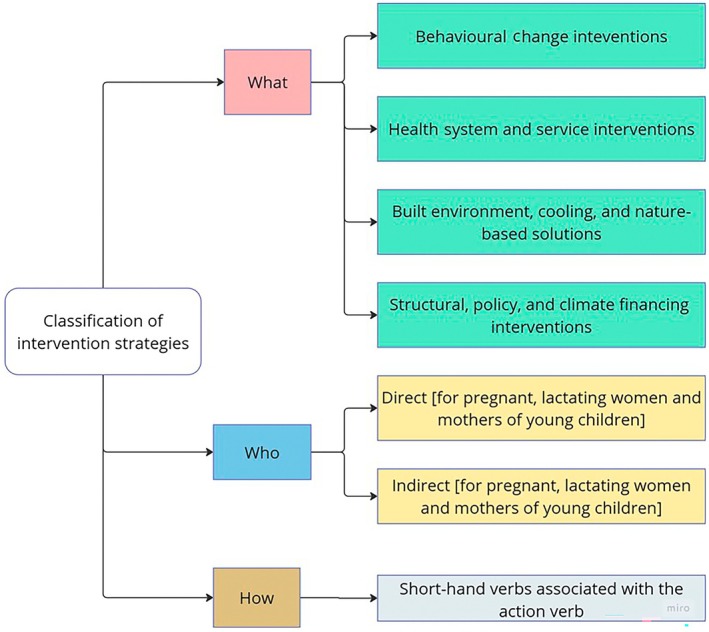
The “what,” “who,” and “how” classification of intervention strategies.

We categorized each strategy according to three guiding questions: (i) What type of strategy is it? (ii) Who is it for? and (iii) How is it intended to work? (Figure 1). To define the strategy type (“what”), we applied the heat adaptation framework of Chersich et al., which outlines four domains: (a) behavioral changes; (b) health system inputs; (c) built environment modifications (particularly cooling); and (d) structural and policy interventions.^15^ Evidence from high‐income settings shows that combining actions across these domains can significantly reduce heat‐related morbidity and mortality.^16^ The “who” was classified as strategies directly targeting pregnant women, lactating women, or women with young children or indirectly including them within broader vulnerable groups. We also recorded the responsible actors (e.g., government agencies, civil society, and community groups) or assigned responsibility to the action plan if unspecified. To assess “how” strategies were designed to function, we identified action verbs (e.g., educate, provide, and communicate), mapped them to the Chersich et al. framework, and tallied their frequency across states/UTs to identify dominant intervention modes and cross‐state trends. Finally, we conducted a thematic analysis to capture common adaptation strategies and identify additional interventions beyond the Chersich et al. framework.

## 3 RESULTS

**FIGURE 2:**
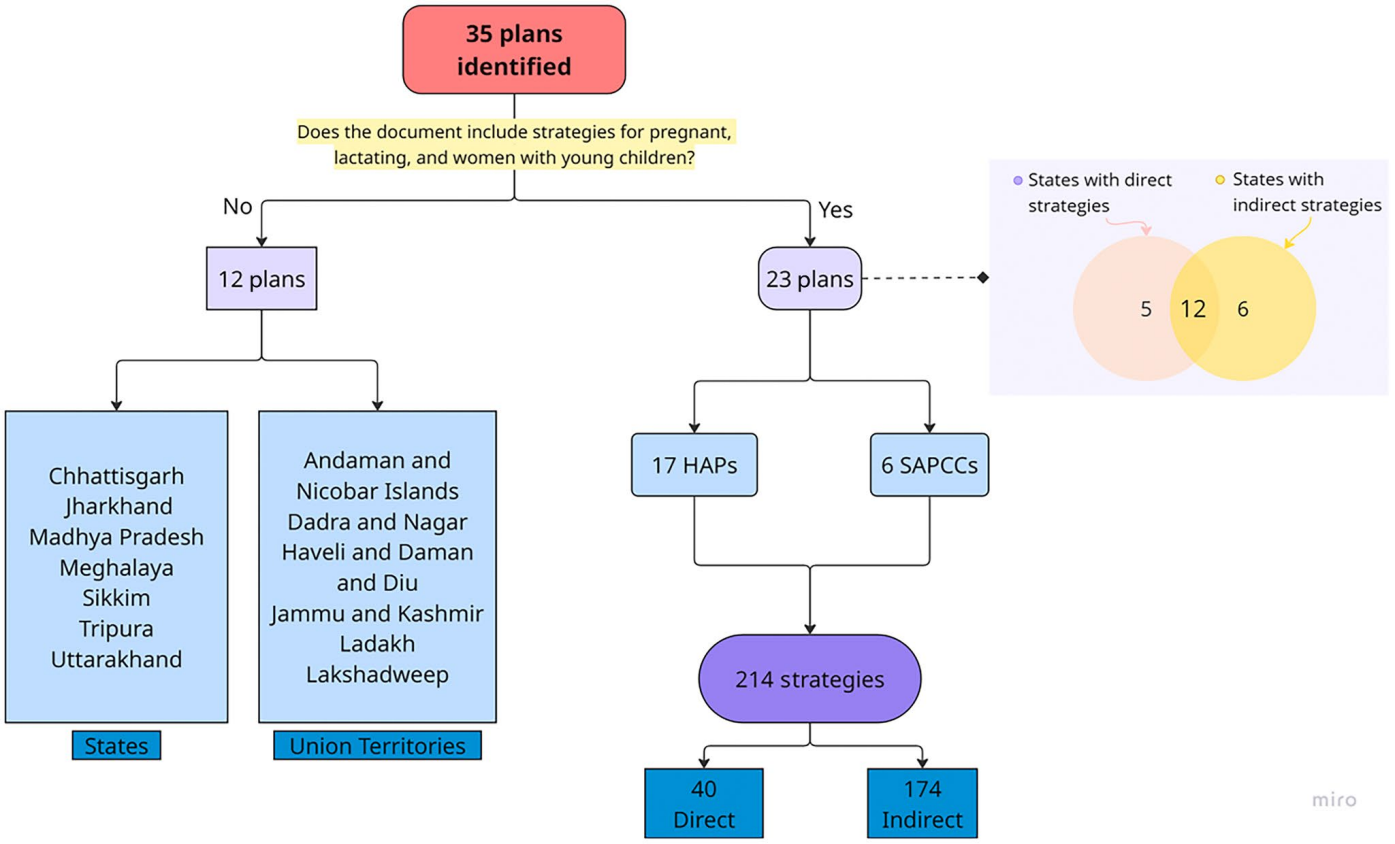
Distribution of intervention strategies from identified document sources. HAPs, Heat Action Plans; SAPCC, State Action Plans for Climate Change.

Of India's 36 states and UTs, we identified 35 climate adaptation plans covering all regions (Chandigarh is included within the Punjab and Haryana documents). These comprised 19 HAPs and 16 SAPCCs.^15^, ^16^, ^17^, ^18^, ^19^, ^20^, ^21^, ^22^, ^23^, ^24^, ^25^, ^26^, ^27^, ^28^, ^29^, ^30^, ^31^, ^32^, ^33^, ^34^, ^35^, ^36^, ^37^, ^38^, ^39^, ^40^, ^41^, ^42^, ^43^, ^44^, ^45^, ^46^, ^47^, ^48^, ^49^, ^50^ Twelve plans (33%) did not include any recommendations for pregnant women, lactating women, or women with young children. The remaining 23 plans featured heat adaptation strategies that targeted this group directly only (five plans), indirectly only (six plans), and both approaches (12 plans), as Figure 2 shows.

**FIGURE 3:**
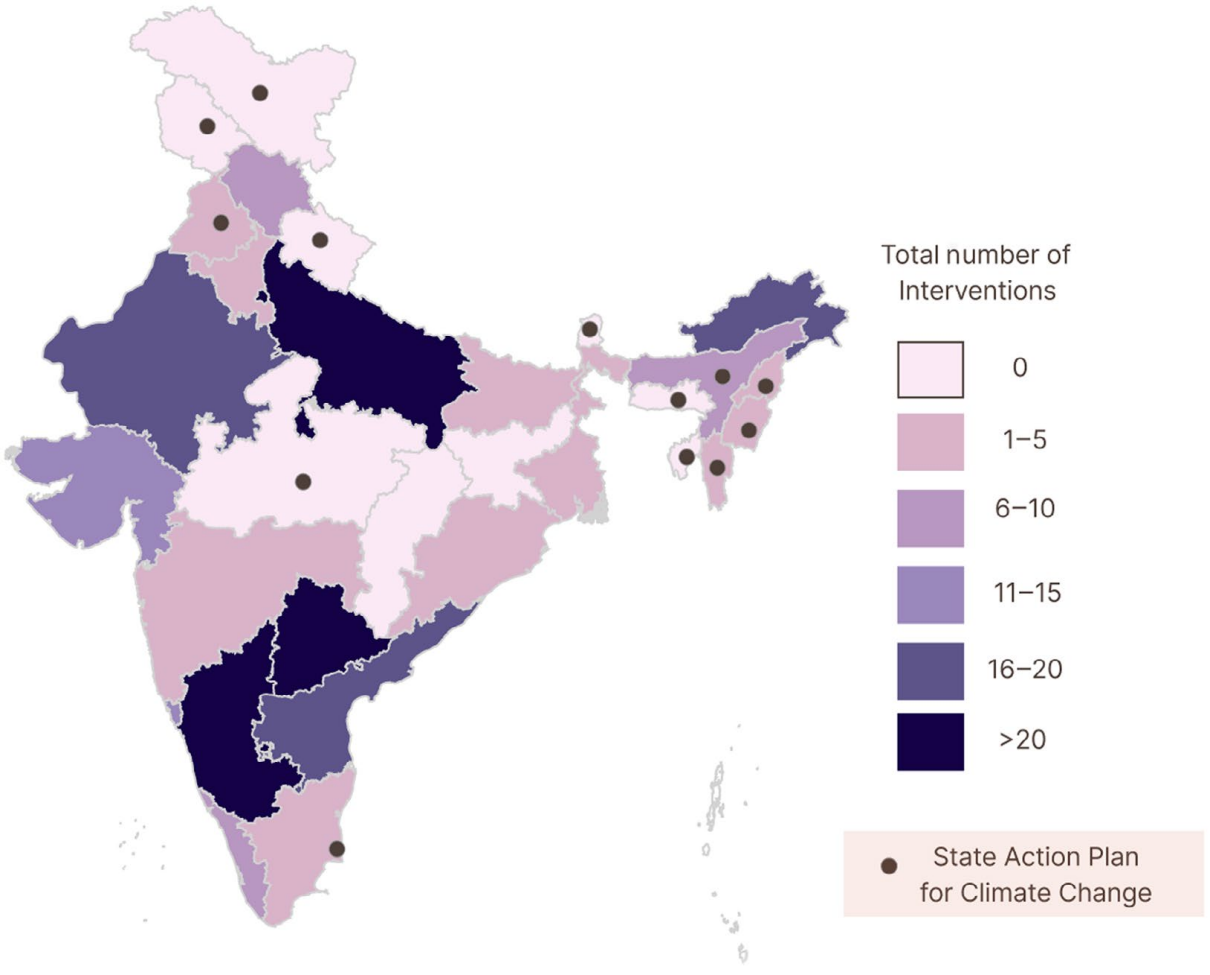
Map of Heat Action Plans (HAPs) and State Action Plans for Climate Change (SAPCCs) with a color gradient to show the number of climate‐related strategies proposed by each state and UT. Darker shades represent a higher number of documented interventions.

Across the 23 plans that included at least one heat adaptation recommendation for the target population, we identified 214 strategies, of which 102 were unique (ranging from 1 to 7 in SAPCCs and 1 to 26 in HAPs) (Figure 3). Of these, 40 strategies directly targeted pregnant, lactating women or women with young children, while 174 addressed them indirectly as part of broader vulnerable groups. Six plans included only indirect measures.

The states with the highest number of unique strategies were Karnataka (*N* = 16), Telangana (*N* = 11), and Delhi National Capital Region (NCR) (*N* = 10).^15^, ^16^, ^17^ Common recommendations included vulnerability assessments, occupational support, and training frontline workers to respond to extreme heat, with particular focus on pregnant women, children, and the elderly.

### 3.1 Intervention strategies: the what, who, and how

**FIGURE 4:**
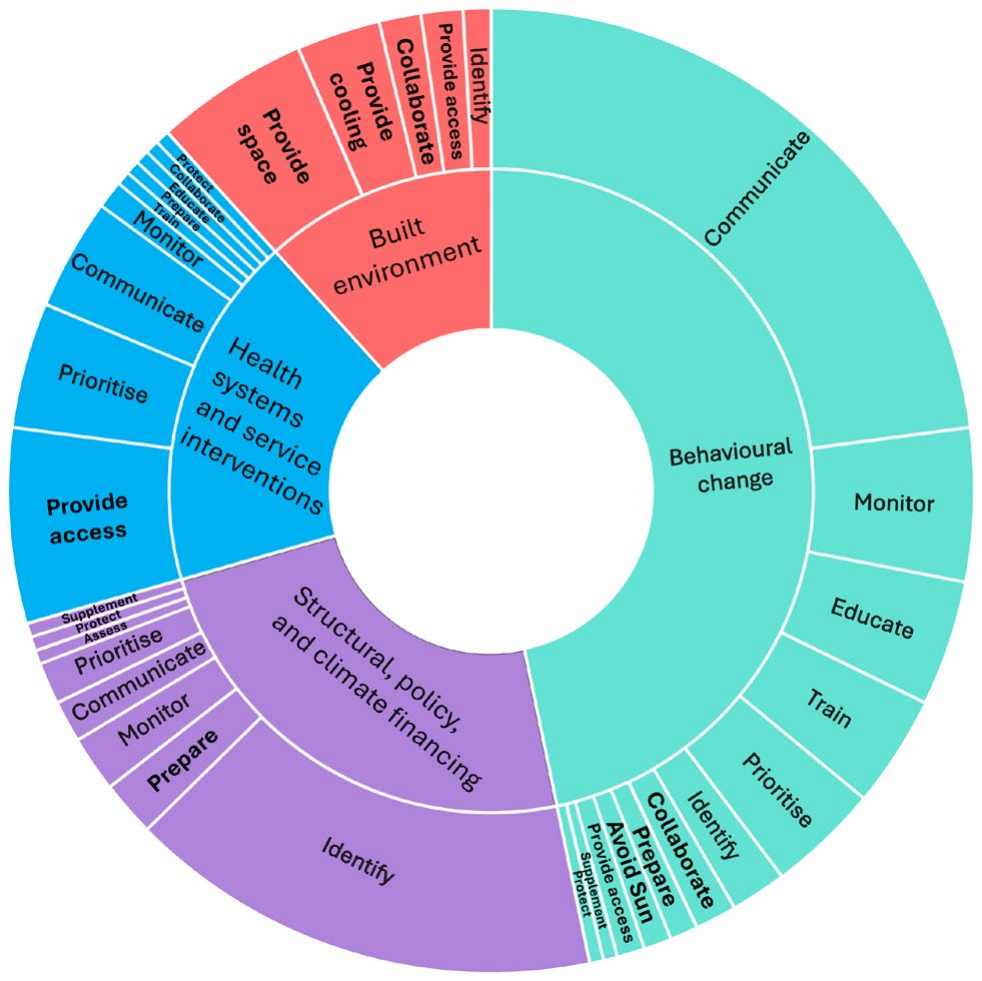
Action verbs grouped by domain based on the Chersich et al. framework. Segment size reflects the number of times each verb was mentioned.

When mapped against the Chersich et al. framework, we obtained 100 behavioral change strategies and fewer built environment (*N* = 25), health system and service interventions (*N* = 38), and structural, policy, and climate financing interventions (*N* = 51). The documents contained a range of action verbs associated with each strategy (Figure 4). Figure S1 illustrates an example of the “what,” “who,” and “how” data extraction from a behavioral change intervention.

#### 3.1.1 Behavioral change

Behavioral change emerged as the predominant strategy for protecting pregnant women, lactating mothers, and those with young children. These interventions featured 15 HAPs and six SAPCCs (*N* = 100), representing 25 of the 40 direct strategies (62.5%) explicitly targeting our population of interest. These included heat protection and dehydration prevention communications for pregnant and nursing women (Rajasthan and Arunachal Pradesh); educational programs for pregnant women, vulnerable groups, and frontline health workers on heat‐related risks (Uttar Pradesh, Haryana, and Telangana); and prioritization protocols for high‐risk patients, including pregnant women, with heat‐related illness (Goa, Karnataka, and Odisha).^15^, ^18^, ^21^, ^25^, ^26^, ^27^, ^32^

When we examined indirect behavioral strategies (*N* = 75), communication and monitoring were the main actions (*N* = 46). These included culturally tailored messaging using local languages and visual aids (Uttar Pradesh, Rajasthan, and Andhra Pradesh)^25^, ^29^, ^31^; practical behavioral guidance on avoiding peak heat exposure, maintaining hydration, and appropriate clothing choices (Kerala, Odisha, and Bihar)^15^, ^22^, ^24^; community outreach programs through Anganwadi workers and self‐help groups across six states (Uttar Pradesh, Telangana, Rajasthan, Delhi, Goa, and Andhra Pradesh)^16^, ^25^, ^27^, ^29^, ^30^, ^31^; workplace protection initiatives such as rescheduling work hours across (Karnataka, Telangana, and Delhi).^25^, ^32^, ^49^ Rajasthan's HAP additionally proposed capacity‐building initiatives for Anganwadi and Accredited Social Health Activists (ASHA) workers, reflecting a trend in other state plans.^25^

#### 3.1.2 Built environment and cooling measures

Built environment and cooling measures was the least mentioned strategy category for protecting pregnant women, lactating mothers, and those with young children across the states/UTs. Built environment strategies were identified in 10 HAPs and zero SAPCCs and represented 25 of the 214 (11.7%) strategies identified in the analysis. We identified only one direct strategy. Telangana's HAP described the provision of safe water and heat protection measures for pregnant women through the Municipal Administration and Urban Development Department.^32^ The remaining 24 indirect strategies identified focused primarily on shelter provision and cooling interventions for vulnerable populations.

Providing space (*N* = 11), cooling (*N* = 5), and access (*N* = 3) were the most frequently identified actions. Examples included mobile cooling van deployment for moving vulnerable groups to cooling centers coordinated through Gujarat's Social Justice and Empowerment Department; expanded access to shaded areas for outdoor workers and slum communities managed by Delhi's Municipal Corporation Climate Change Nodal Officer; ice pack dispensary creation across seven states (Uttar Pradesh, Gujarat, Telangana, Goa, Rajasthan, Andhra Pradesh, and Delhi)^16^, ^18^, ^20^, ^21^, ^27^, ^29^, ^32^; and safe drinking water provision for farmers, slum dwellers, and vulnerable communities through Telangana's Rural Water Supply and Sanitation Department.^32^

#### 3.1.3 Health system and service interventions

Health system and service interventions were identified in 14 HAPs and 1 SAPCC (*N* = 38), with 10 direct strategies (26%) and 28 indirect strategies (74%).

Prioritizing (*N* = 8), monitoring (*N* = 1), and protecting (*N* = 1) were the actions identified in the direct strategies. These included measures to ensure pregnant workers and workers with medical conditions receive additional attention across eight states (West Bengal, Uttar Pradesh, Tamil Nadu, Odisha, Karnataka, Himachal Pradesh, Gujarat, and Telangana) coordinated through Departments of Health and Family Welfare with employer assistance.^20^, ^22^, ^25^, ^26^, ^28^, ^30^, ^31^, ^32^ Additionally, The Goan Women and Child Development Department implemented surveillance mechanisms to create tracking systems for children, lactating mothers, and women through Integrated Child Development Scheme (ICDS) and Anganwadi centers.^18^ Finally, Maharashtra entrusted their District Disaster Management Authorities to ensure protection from the effects of heat for pregnant women during a heat wave event.^50^

The indirect strategies included provision of access and communication, accounting for 14 and eight strategies, respectively. Some of these approaches included establishing outreach clinics at accessible locations (Uttar Pradesh, Goa, Karnataka, Himachal Pradesh, Gujarat, Telangana, Rajasthan, Arunachal Pradesh, Mizoram and Delhi),^15^, ^18^, ^20^, ^22^, ^25^, ^27^, ^29^, ^31^, ^32^, ^40^ coordinated through directors and in‐charges of hospitals, Community Health Centres, Primary Care Health Centres, and Urban Health Centres. Seven states detailed heat‐related illness prevention communications (dos and don'ts) using various media platforms under the same healthcare administrative structure and spearheaded by the Family and Welfare Department representatives and hospital directors of the different states and districts (Uttar Pradesh, Karnataka, Himachal Pradesh, Gujarat, Goa, Rajasthan, and Arunachal Pradesh).^15^, ^18^, ^20^, ^22^, ^25^, ^27^, ^31^

#### 3.1.4 Structural, policy, and climate financing

We identified these strategies in 10 HAPs and zero SAPCCs (*N* = 51), representing 24% of total strategies. Of these, four were direct strategies, including three strategies where a surveillance system was created for monitoring and tracking lactating mothers among other groups (Uttar Pradesh, Telangana, and Rajasthan),^27^, ^31^, ^32^ coordinated through Urban Development and Panchayati Raj Departments; and heatwave impact monitoring and evaluation on infants, women, and lactating mothers managed by Telangana's Women Development and Child Development Department.^32^ The remaining 47 indirect strategies focused primarily on risk identification and system preparedness for vulnerable populations.

Identification (*N* = 34) and preparation (*N* = 4) were the main actions in the indirect strategies, with identification focused on at‐risk populations and high‐heat areas and preparation encompassing vulnerability assessments, risk mapping, and development of precautionary measures for vulnerable groups. These included vulnerability mapping with non‐governmental organization (NGO) involvement across Karnataka, Uttar Pradesh, Rajasthan, Andhra Pradesh, and Delhi coordinated through Revenue Departments^16^, ^25^, ^27^, ^29^, ^31^; heat‐sensitive planning integration into City Development Plans managed by Delhi's District Disaster Management Authority^29^; databank creation and hazard risk mapping implemented by Karnataka's Revenue Department and State Natural Disaster Monitoring Centre^25^; and power management policies for heat adaptation developed by Gujarat's Energy and Petrochemicals Department and Arunachal Pradesh's Power Department.^15^, ^20^

## 4 DISCUSSION

Our India‐wide analysis of 35 policy documents reveals a diverse set of state‐level commitments and actions aimed at protecting pregnant women, lactating mothers, and women with young children from extreme heat. In the global context, India stands out for having published more HAPs than any other low‐ and middle‐income country.^51^ To understand how India's approach compares internationally, we drew upon a recent WHO‐commissioned scoping review that analyzed how maternal, newborn, and child health (MNCH) is addressed in Heat‐Health Action Plans globally, including 35 plans from Indian states and cities.^14^ The scoping review confirmed that Indian HAPs more frequently identified pregnant women and children as vulnerable groups compared to other countries. Specifically, 60% of Indian HAPs recognized pregnant women as vulnerable populations versus 46% in non‐Indian HAPs. However, it also highlighted key gaps in the Indian context, including the absence of defined indicators to monitor plan effectiveness and repeated content across state plans, patterns mirrored in our analysis. While offering valuable global comparisons, it did not examine the detailed strategies, implementation pathways, or specific mechanisms addressing pregnant and lactating women within Indian plans.

Our study provides a deeper, India‐specific analysis of all states and UTs. We move beyond cataloguing strategies to examine how they are proposed to be implemented, their alignment with evidence‐based frameworks, and variations across state contexts. By categorizing strategies as direct or indirect using a climate‐focused conceptual framework, we illustrate how these approaches extend beyond the traditional “identify–prepare–respond–recover” model. We further situate India's approaches within international best practices, highlighting both its distinctiveness and the innovative models emerging domestically that could inform national scaling and global adaptation efforts.

Our comprehensive analysis identified 214 heat adaptation strategies across Indian HAPs, revealing important patterns in how states address vulnerable populations. Notably, fewer than 40 (19%) explicitly addressed the needs of pregnant and lactating women. This is important, given the emerging epidemiological evidence documenting heightened physiological susceptibility among pregnant and lactating women to heat‐related morbidity.^52^, ^53^, ^54^ However, a more nuanced picture emerges when indirect strategies are considered. A majority, 174 strategies (81%), focused on broader categories of vulnerable populations, within which pregnant and lactating women are implicitly included. This pattern suggests a recognition of intersectional vulnerabilities, although it also reflects missed opportunities for more targeted, population‐specific interventions. The variation in policy attention across states highlights key differences in how state governments are shaping their heat adaptation responses. Each state's approach reflects unique governance contexts, resource availability, and institutional capacities, together offering a spectrum of adaptation models and valuable lessons for broader replication.

To understand how these strategies could translate into practice, our analysis revealed that states across India are adopting distinct models of heat adaptation, each shaped by local governance structures, institutional capacities, and external support. For example, Karnataka presents a technical capacity‐building model enabled through strategic public–private partnerships.^25^ With support from the Asian Development Bank, the state implemented 16 distinct strategies spanning all four core adaptation categories, with a particular emphasis on “identify” and “prepare” actions. These include the integration of heat‐adaptive measures into social protection programs and the deployment of mobile health clinics and water kiosks in informal work zones. Karnataka's approach illustrates how external technical and financial support can enable more sophisticated and context‐responsive adaptation planning.

In contrast, Telangana's health system‐centered approach represents a different pathway that leverages existing health infrastructure to deliver targeted interventions.^32^ With 26 total strategies focused on medical personnel across Community Health Centres, Primary Health Centres, and Urban Health Centres, Telangana demonstrates how embedding climate adaptation within established service delivery systems can achieve comprehensive responses. By integrating direct measures like monitoring pregnant and lactating women through the ICDS systems with indirect interventions such as mobile health clinics, this model offers particularly valuable insights for contexts where health systems are relatively well developed and can serve as the institutional cornerstone for adaptation efforts.

Delhi NCR's structural and systemic approach presents a third model centered on multi‐stakeholder coordination and rapid response capabilities.^29^ With 10 unique strategies focused on risk mapping and coordinated response, the state emphasizes “identify” and “prepare” actions coordinated through networks involving the India Meteorological Department, Municipal Corporation, Delhi Police, ASHAs, and NGOs. This collaborative governance model demonstrates how climate adaptation can be embedded within broader urban governance systems, suggesting that in complex urban contexts, effective adaptation might depend on the ability to coordinate diverse actors during crisis response.

These different approaches are examples demonstrating that effective heat adaptation for vulnerable populations is not dependent on a single “best practice” model but rather on how well adaptation strategies align with existing institutional strengths and governance contexts.

Our findings reveal that while one‐third of plans contained no considerations for pregnant and lactating women, those that do mostly focus on behavioral change (short‐to‐medium term action) and policy strategies (long‐term actions). However, this predominance of behavioral interventions in India contrasts with the existing evidence on intervention effectiveness for protecting pregnant and lactating women from extreme heat.

The comprehensive framework of Chersich et al. requires a balanced approach across all four intervention categories, with particular emphasis on built environment modifications and health system strengthening for physiologically and socially vulnerable populations.^55^ While behavioral interventions are effective and widely adopted, they cannot address the deeper systemic vulnerabilities that require structural and policy reforms. The prominence of behavioral change strategies in the Indian state plans reflects a limited recognition of the structural determinants of heat vulnerability among pregnant and lactating women.^56^

A systematic review on gendered vulnerability and climate change argued that focusing on individual or community‐level responses often overlooks the structural inequalities like poverty, insecure work, and inadequate housing, which prevent women and other marginalized groups from meaningfully adapting to climate risks.^57^ This is worsened by the persistent underrepresentation of gender‐disaggregated health data in climate change research and policy development.^4^ This data gap must alert health professionals and policymakers to the specific challenges facing women and girls, particularly as climate change threatens to deepen existing social and economic inequalities.

A common thread across all state models is the significant role of community‐based intervention strategies, which constitute a substantial proportion of identified policy approaches. These approaches leverage existing healthcare and social service networks, which include community health workers (CHWs), Self Employed Women's Association members, and ICDS functionaries, as evidenced in strategies found across states like Uttar Pradesh, Telangana, Rajasthan, Delhi, Goa, and Andhra Pradesh.^18^, ^27^, ^29^, ^31^, ^32^ The community interventions encompass multiple approaches: capacity building and training for heat protection (Gujarat, Telangana, Goa, and Arunachal Pradesh), implementing public awareness campaigns (Uttar Pradesh, Rajasthan, Karnataka, Himachal Pradesh, Gujarat, Goa, and Arunachal Pradesh) and utilizing SMS text messages as a non‐traditional form of information dissemination (Uttar Pradesh, Rajasthan, and Andhra Pradesh).

This focus on community‐based interventions aligns with other international plans of heat adaptation. The global scoping review on MNCH found that “informing, educating and awareness raising” was the most common strategy, appearing in 49 out of 83 HAPs examined. International evidence supports the effectiveness of these approaches. In Pakistan, the role of CHWs was crucial in a randomized controlled trial study focused on elaborating tailored health messages to the community.^58^ They observed a 38% reduction in unscheduled hospital visits with improved heat‐health literacy and practices. Similarly, in Dhangadhi, Nepal, the Female Community Health Volunteers conduct awareness campaigns by disseminating heat‐health information to pregnant and lactating women and other vulnerable populations, demonstrating their key role in community protection.^59^

However, research from high‐income countries with established heat‐health programs provides stronger evidence for more comprehensive approaches. For example, Australia's National Heatwave Warning Framework and France's Heatwave action plan response system demonstrate that countries achieving significant reductions in heat‐related maternal and neonatal morbidity prioritize structural interventions and healthcare system preparedness.^60^, ^61^ Similarly, evidence from Italy and Germany demonstrated that comprehensive heat‐health action plans incorporating multi‐sectoral and multiple intervention strategies can effectively improve measurable heat‐related health outcomes and reduce mortality.^62^ This international evidence shows that comprehensive approaches produce more sustainable outcomes than awareness‐based interventions alone, corroborating the comprehensive framework of Chersich et al. for adaptation.

### 4.1 Implications for policy

Our findings have implications for climate health policy development in India and similar contexts. The current intervention mix suggests an urgent need to rebalance heat adaptation planning toward evidence‐based structural measures that address the systemic vulnerabilities of pregnant and lactating women. Priority should be given to sustainable financing for comprehensive structural interventions, including cooling infrastructure in maternal healthcare facilities, occupational safety regulations for pregnant workers, and built environment modifications in high‐risk communities. The variation in state‐level approaches identified in this analysis with Karnataka, Telangana, and Delhi NCR demonstrates that more comprehensive strategies might provide valuable models for scaling effective interventions nationally.^25^, ^29^, ^32^ However, achieving meaningful protection for India's most heat‐vulnerable populations will require moving beyond the current emphasis on community‐based behavioral change toward systematic investment in the structural determinants of heat resilience.

Drawing on the Oxford Policy Management (2017) review of India's State Action Plans on Climate Change, we recommend broadening intervention portfolios beyond the current overreliance on communication‐based strategies.^63^ States should shift from primarily “communicate” and “educate” actions to include more “provide,” “protect,” and “supplement” measures, especially within built environment and health system categories, where such interventions remain limited. With only 19% of strategies directly addressing pregnant and lactating women, there is clear potential to convert indirect approaches into direct ones, as demonstrated by states like Telangana and Karnataka across all intervention categories.^25^, ^32^

Achieving this shift will require not only increased domestic climate financing but also institutional reforms that enable long‐term, evidence‐informed planning, rather than the reactive, awareness‐focused models. Effective adaptation for vulnerable women demands not just innovation but integration by building on diverse state‐level strengths while tackling the structural barriers that undermine their impact.

### 4.2 Implications for research

Significant knowledge gaps remain regarding the physiological impacts of extreme heat on pregnant and lactating women in the Indian context, warranting urgent research attention. Ongoing epidemiological studies will be essential to inform the scope and specificity of the HAP interventions and broader climate adaptation policies. This evidence base is critical for developing targeted, evidence‐informed policy responses.

For example, Tamil Nadu's initiative to compensate families of heatwave victims and relief workers could be expanded to include pregnant and lactating women.^28^ Additionally, research into the lived experiences of these women during extreme heat events can uncover feasible coping strategies. If found effective, such strategies could be supported, scaled through HAPs SAPCCs, and ensure that women's perspectives are reflected in policy. Comparative studies across states with varied HAP approaches could further identify best practices.

Our findings underscore the urgent need for more explicit integration of pregnant and lactating women into HAPs and national adaptation strategies. While many plans broadly reference “vulnerable groups,” specific interventions for this population are lacking (12 states and UTs had no such measures). These states require prioritized engagement through advocacy, technical support, and follow‐up, drawing on examples from Karnataka and Ananthapuramu.^25^, ^64^

Finally, the analysis shows that many HAPs and SAPCCs lack region‐specific contextualization, often replicating generic templates rather than tailoring strategies to local risk profiles. This highlights the need for localized vulnerability assessments and evidence‐based planning to effectively address geographically and culturally specific heat‐health risks.

### 4.3 Limitations

Our access to HAPs was restricted, as documents from some states were not publicly available or accessible through our sources. Additionally, while HAPs and SAPCCs included universal strategies that could indirectly benefit pregnant and lactating women, our analysis focused only on explicitly targeted measures. This approach might have excluded relevant provisions under broader categories (e.g., workers and caregivers), potentially overlooking existing but non‐specific interventions. Language barriers were also present.

## 5 CONCLUSION

Our review of HAPs and SAPCCs identified over 200 strategies, including over 100 unique ones, of which 40 directly targeted pregnant and/or lactating women. Most strategies were focused on behavioral change, while some unique strategies and arrangements were also identified in particular states. Strategies specifically addressing the needs of pregnant and lactating women during extreme heat events remain limited, likely reflecting a broader lack of understanding of the target group's particular vulnerabilities to extreme heat and lack of evidence for targeted interventions. There were no strategies in 12 states and union territories, suggesting critical gaps in adaptation planning. It is imperative to assess and strengthen targeted and universal policies and implement concrete, action‐oriented adaptation measures.

## AUTHOR CONTRIBUTIONS

JH and DN contributed to the conceptualization of the paper. GDJCF and SS conducted the research. GDJCF, SS, and KK screened and extracted data. GDJCF and SS conducted the data analysis. SS prepared the original manuscript. JH and DN reviewed and edited initial drafts. GDJCF prepared the final manuscript. All authors were involved in critically reviewing and approving the final manuscript.

## CONFLICT OF INTEREST STATEMENT

The authors have no conflicts of interest to declare.

## Supporting information


Figure S1.

## Data Availability

The data that supports the findings of this study are available in public networks. Direct links to the documents are provided in the reference list. In instances where plans were not accessible through official websites, they were obtained via relevant professional and research networks and are cited accordingly.
