# Supplementary material for: Safeguarding maternal health against extreme heat in India: A policy analysis of state‐level heat action plans
Source: Int J Gynaecol Obstet. 2025 Dec 17;173(3):1443–53. doi: 10.1002/ijgo.70747 (PMC13173611; doi:10.1002/ijgo.70747)
Supplement: Supplementary file 1 — Figure S1. [file IJGO-173-1443-s001.docx]

Supplementary file 1


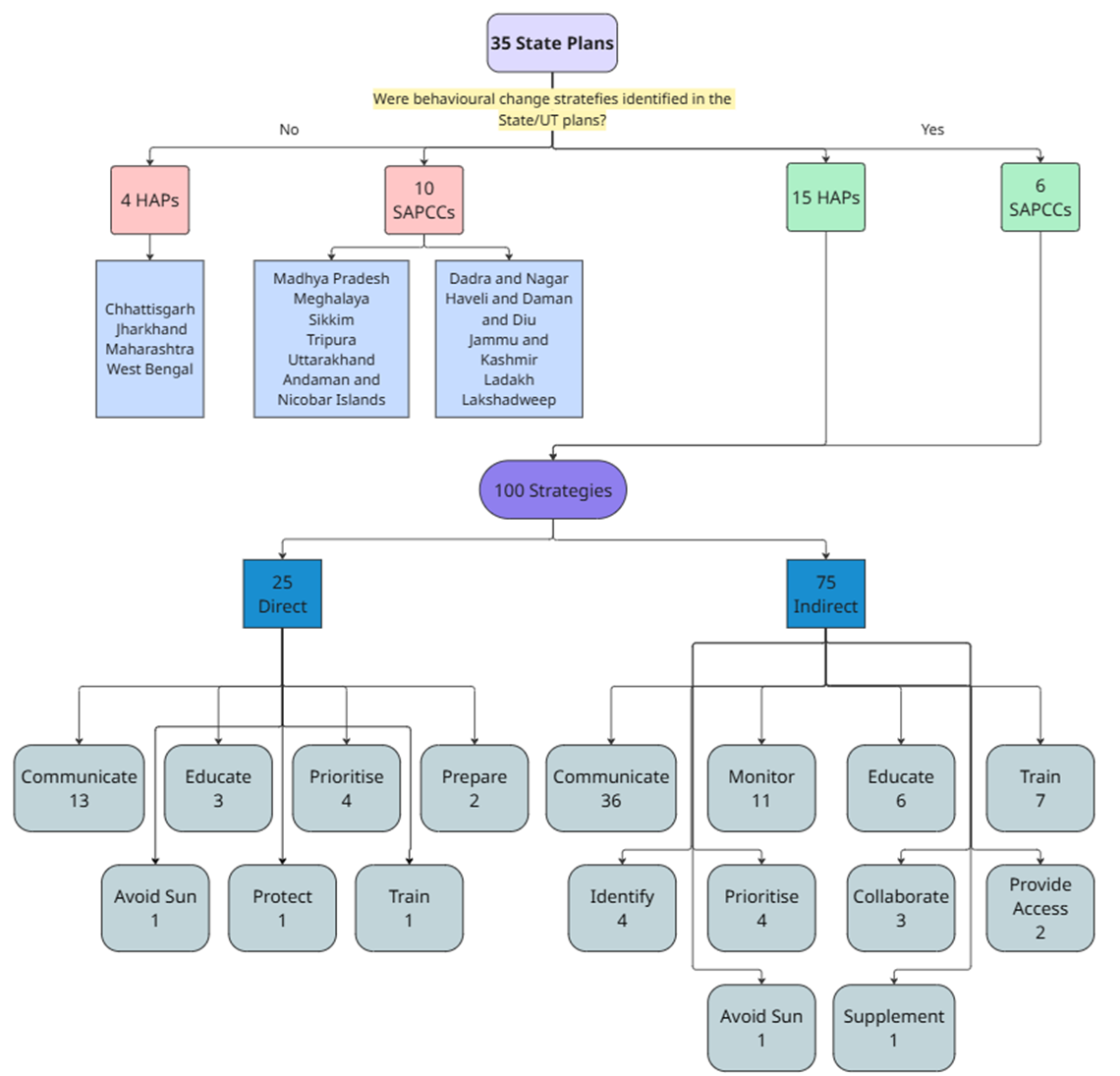


***Figure*** S1 *Illustrative Example of ‘What’, ‘Who’, and ‘How’ Coding for Behavioural Change Interventions*
